# A Banquet without Wine

**Published:** 1899-12

**Authors:** 


### A Banquet Without Wine.

The Wyoming State Medical Society has recently set an example which might be followed with advantage by some other State medical bodies of the Pacific coast. This society had what is reported to be the most interesting banquet which has ever been held in that State and without the use of alcoholics at the feast. We notice that there is a disposition, and we are glad to see it, to dispense with wines at these meetings. As the views of numerous members of these societies are severely tried on account of liquors at these meetings, if it shall be decided that a successful banquet can be had without them, much will have been gained. The *Sentinel* congratulates the Wyoming State Medical Society on its courage, energy, enterprise and progressiveness in this matter.—*The Medical Sentinel.*

